# Young Spelt Grass Juice as a Functional Food: Therapeutic Potential and Phytochemical Composition

**DOI:** 10.3390/foods15213957

**Published:** 2026-11-04

**Authors:** Biljana Milovanovic, Marina Nikolic, Milos Nikolic, Marijana Andjic, Nevena Lazarevic, Vladimir Jakovljevic

**Affiliations:** 1 Center of Excellence for Redox Balance Research in Cardiovascular and Metabolic Disorders, 34 000 Kragujevac, Serbia; biljalazovic97@gmail.com (B.M.); andjicmarijana10@gmail.com (M.A.); nevenasdraginic@gmail.com (N.L.); vladimir.jakovljevic@fmn.kg.ac.rs (V.J.); 2 Department of Physiology, Faculty of Medical Sciences, University of Kragujevac, 34 000 Kragujevac, Serbia; 3 Department of Pharmacy, Faculty of Medical Sciences, University of Kragujevac, 34 000 Kragujevac, Serbia; milos.nikolic@fmn.kg.ac.rs; 4 Department of Pathophysiology, 1st Moscow State Medical, University IM Sechenov, Moscow 119146, Russia; 5 Faculty of Medicine, University of Banja Luka, 78000 Banja Luka, Bosnia and Herzegovina

**Keywords:** spelt grass juice, bioactive compounds, phenolic acids, functional nutrition, antioxidant activity, oxidative stress

## Abstract

Spelt (*Triticum spelta* L.) and its products, including young grass and juice, attract increasing attention due to their nutritional and phytochemical composition. However, spelt juice remains relatively understudied as a functional food. This review summarizes the available evidence on its phytochemical composition, health-promising activities, and factors influencing product quality. Its composition depends on growing conditions, harvesting time, and processing methods. Major bioactive constituents include phenolic acids, flavonoids, chlorophylls, and carotenoids, with ferulic acid being particularly abundant. These compounds may contribute to antioxidant and anti-inflammatory activities and the modulation of metabolic processes. Preliminary human findings suggest that spelt juice consumption may improve selected biomarkers of oxidative stress, antioxidant status, and inflammation. Nevertheless, evidence specifically addressing spelt grass juice remains limited, with much of the available data derived from in vitro studies and research on spelt seeds, extracts, or young grass. Further preclinical and clinical studies are needed to clarify its biological effects, mechanisms of action, and potential health benefits. Overall, young spelt grass juice represents a promising functional food, but its proposed therapeutic effects require confirmation through well-designed experimental and clinical studies.

## 1. Introduction

Functional foods represent one of the key areas of modern nutrition science. In addition to basic nutritional value, they contain naturally occurring or added bioactive compounds that contribute to health maintenance and disease prevention [1]. Over the past few decades, functional foods have attracted considerable attention from both researchers and public health professionals due to accumulating evidence of their beneficial biological effects [2]. A growing body of preclinical and clinical evidence indicates that functional foods exert beneficial effects on human health by positively influencing physiological processes, supporting overall well-being, and reducing the risk of chronic diseases, particularly cardiovascular, neurological, gastrointestinal, and malignant disorders [1,3]. Numerous foods derived from both plant and animal origin, such as fruits, vegetables, dairy products, meat, fish, and cereals, contain biologically active substances that may contribute to these health-promoting effects [4].

Cereals are essential food products consumed worldwide and constitute an important source of micro- and macronutrients, carbohydrates, proteins, dietary fiber, minerals, and vitamins [5]. Various studies have shown that regular consumption of whole grains reduced the risk of chronic diseases such as type 2 diabetes, obesity, cardiovascular diseases, and cancer, as well as mortality resulting from the progression of these diseases [6,7]. Particular emphasis has been placed on ancient grains, which represent a good source of phytochemicals and exhibit antioxidant, anti-inflammatory, cardio-protective, and immunomodulatory effects [8]. Indeed, these health benefits cannot be extrapolated to refined grains, as the refining process substantially reduced their content of fiber, vitamins, minerals, and phytochemicals. Although refined cereals provide a significant source of energy, their lower nutritional values and a higher glycemic index may contribute to adverse cardiometabolic outcomes when consumed in excess. Overall, refined cereals represent an important topic of public health due to close correlation of their consumption with cardiometabolic diseases worldwide.

Increasing attention has been directed toward spelt (*Triticum spelta* L.), an ancient wheat species characterized by a rich nutritional and phytochemical composition [9]. Compared with other wheat species, spelt contains higher levels of proteins, minerals, lipids, a more favorable fatty acid profile, as well as other bioactive components such as phenolics and flavonoids [10]. Spelt has multiple beneficial effects on health and its regular consumption can lead to a decrease in glucose, insulin, and blood lipid levels, as well as a reduction in inflammatory processes in the body and the risk of developing chronic diseases [11].

Spelt grain has traditionally been used in human nutrition and in the production of functional foods. However, in recent years, there has been growing interest in organic products, with increasing attention toward young spelt grass, and juice obtained by pressing [12]. Additionally, increased interest in this natural products consumption may be attributed to their phytochemical composition including numerous bioactive components, including amino acids, flavonoids, vitamins, minerals, chlorophyll, and other phytochemicals, and has been associated with antioxidant, anti-inflammatory, immunomodulatory, and detoxifying effects [13].

While previous reviews have examined the nutritional properties of ancient wheat species and the potential health effects of wheatgrass, young spelt grass juice has received limited attention in the review literature. Although spelt is an important ancient cereal with valuable nutritional properties, its juice as a functional food requires further investigation due to the limited availability of data. Also, the lack of standardization in juice production and analysis complicates comparisons between studies [14]. Therefore, the aim of this review article is to provide a detailed analysis of the available literature on young spelt grass juice, with particular emphasis on its phytochemical composition and therapeutic potential, while identifying gaps in the existing literature and directions for future studies.

## 2. Literature Search

This article is a narrative review based primarily on searches of the biomedical literature using PubMed/MEDLINE and Google Scholar. In addition, the reference lists of relevant publications were screened to identify further studies of interest. Certain taxonomic and botanical information was also obtained from relevant botanical sources, including Flora of North America.

The literature search was conducted between June and September 2026. Most of the included publications were published within the last 10 years, although studies published up to 20 years ago were also considered when relevant to the scope of this review. The main search terms included “spelt grass juice”, “Triticum spelta”, “functional food”, “bioactive compounds”, “phenolic acids”, “antioxidant activity”, and “therapeutic potential”. These terms were used individually and in various combinations.

Publications were selected based on their relevance to the objectives of this review and the availability of pertinent information. Studies providing information on the phytochemical composition, antioxidant activity, or potential health effects of young spelt grass juice were included. Studies on spelt grain, young wheatgrass juice, and other cereal grasses were also considered, with findings from these sources clearly distinguished from those specifically related to young spelt grass juice. Publications unrelated to the scope of this review were excluded.

## 3. Botanical and Nutritional Characteristics of Spelt

### 3.1. Botanical and Agronomic Characteristics of Spelt

Spelt (*Triticum spelta* L.), also classified as *Triticum aestivum* ssp. *spelta* L., is a hexaploid cereal (2n = 6x = 42) belonging to the family Poaceae. Its AABBDD genome originated through hybridization between tetraploid emmer wheat (*Triticum turgidum* ssp. dicoccum) and the diploid wild species *Aegilops tauschii* [15]. European and Asian forms of spelt are recognized, with European spelt documented in Central Europe during the Early Bronze Age. During the twentieth century, spelt was largely replaced by common wheat because of its higher yield, better bread-making properties, and lower production costs. However, interest in spelt has recently increased due to its nutritional composition and potential functional properties [16,17].

Spelt is a tall annual grass, typically reaching 100–150 cm, with long spikes and grains enclosed in persistent hulls [18,19,20]. Unlike common wheat, its grains remain covered by glumes after threshing, providing mechanical protection against environmental contamination and pathogens [21]. The grain consists primarily of endosperm (80–85%), bran (13–19%), and germ (approximately 3%) [18].

Spelt is valued for its adaptability to diverse environmental conditions and its suitability for traditional and organic farming. Its hull may contribute to protection against stress and fungal diseases, potentially reducing the need for agricultural inputs [12,22,23,24]. However, compared to modern bread wheat varieties, spelt generally has lower yields (for 37% compared to bread wheat), is more susceptible to lodging due to its tall stature (approximately 30 cm taller), and requires an additional dehulling step, which increases processing demands and may result in grain losses [25].

### 3.2. Nutritional Characteristics of Spelt

The high nutritional potential of spelt is reflected in its rich content of macronutrients, micronutrients, and bioactive components, whose content depends on environmental factors, genotype, and cultivation conditions [26].

Proteins represent one of the most important components of spelt. Several protein fractions are present in spelt grain, including gluten proteins, composed of glutenins and gliadins, which constitute the largest proportion of grain proteins and serve as amino acid reserves during germination, as well as albumins and globulins [27]. Spelt proteins contain relatively low proportions of essential amino acids such as lysine and threonine, and higher levels of glutamine and proline [28]. Carbohydrates are the main energy source in spelt grain, predominantly in the form of starch, while its dietary fiber, including arabinoxylans and β-glucans, may contribute to gastrointestinal health and the regulation of glucose and lipid metabolism [26,29,30]. Spelt lipids consist mainly of unsaturated fatty acids, particularly linoleic acid [31,32].

The nutritional composition of spelt has been compared with that of common wheat, with studies reporting differences in protein, lipid, and dietary fiber contents, as well as in selected mineral constituents. Some studies have reported higher protein and lipid contents in spelt, highlighting its potential as a nutritionally valuable cereal. A comparison of selected nutritional characteristics of spelt and common wheat is presented in Table 1. The reported values originate from different studies and may vary depending on genotype, growing conditions, sample type, and analytical methods.

Spelt also contains minerals, including potassium, magnesium, phosphorus, iron, copper, zinc, and manganese [35]. Their content is higher in whole-grain products because minerals are concentrated mainly in the germ and bran, which are partially removed during milling [36]. The main vitamins are B-group vitamins, particularly thiamine, riboflavin, and niacin, while vitamin E and carotenoids are present in smaller amounts [28].

Although the nutritional, botanical, and agronomic characteristics of spelt grain have been well investigated, considerably less is known about the biochemical composition of young spelt grass and its juice, as well as the changes occurring during germination and early plant development. Consequently, increasing attention has been directed toward the phytochemical composition of young cereals, particularly young spelt grass and spelt juice, and their potential health benefits as functional foods [12].

## 4. Young Spelt Grass and Its Importance in the Production of Spelt Juice

### 4.1. Germination and Early Development of Cereals

Germination and early plant development are accompanied by active metabolic changes that may increase the content of bioactive compounds. Compared with mature cereals, young cereals are characterized by a more favorable nutritional profile, higher amounts of certain nutrients and biologically active components, which are more easily digested and have lower allergenic potential. They contain higher amounts of proteins, vitamins, fibers, phenolic compounds, and flavonoids [34].

During germination, both nutritional quality of cereals and the bioavailability of biologically active compounds are improved, with relatively low costs and good environmental sustainability [37,38,39,40]. Additionally, hydrolytic enzymes are activated, while macronutrients such as carbohydrates, proteins, and fats are degraded, with the release of simple sugars, free amino acids, and fatty acids [41]. Of particular importance are amylases, which break down starch into simpler sugars, thereby releasing the energy necessary for the growth and development of the sprout. In addition to amylases, phytases are also activated, which degrade phytic acid, an antinutritional factor in cereals that binds minerals and re-duces their bioavailability. Phytase hydrolyzes phytic acid into phosphoric acid and myo-inositol, thus making minerals bioavailable [42].

Germination enhances the accumulation of vitamins and secondary metabolites, particularly polyphenols and flavonoids, thereby increasing antioxidant activity and potentially enhancing cellular protection against oxidative stress through multiple physiological mechanisms. These effects may contribute to reduced risk of chronic diseases such as diabetes, cardiovascular diseases, and cancer [43,44,45]. These biochemical changes that occur during cereal germination depend on their genotype and germination conditions, which include the type of cultivation substrate, temperature, humidity, and duration of germination. Moreover, it could influence the phytochemical and nutritional composition of young cereals, implying that the selection of optimal germination conditions is a very important factor in obtaining high-quality young green biomass [45,46].

The results of earlier conducted studies have shown the importance of germination conditions regarding phytochemical accumulation. Additionally, germination at 21 °C for 7 days was identified as optimal for increasing soluble phenolic compounds and antioxidant and anti-inflammatory potential [47]. Moreover, another study reported that wheat grains soaked for 24 h and subsequently germinated at 16.5 °C showed increased concentrations of vitamin C, vitamin E, β-carotene, ferulic acid, and vanillic acid, with several antioxidant components reaching their highest levels after 7 days. Based on these findings, it is indicated that germination duration as well as temperature should be optimized according to the target bioactive compounds. Furthermore, pretreatment before germination may influence phytochemical profile. It was shown that 24-h soaking followed by germination produced a more favorable combination of antioxidant content and sensory properties than prolonged soaking for 48 h [48]. Also, the distribution of phenolic compounds may be altered by germination, but the changes in individual compounds may not occur in the same direction.

In the literature, the term “wheatgrass” refers to young green biomass, primarily the shoots of *Triticum aestivum*, but it may also include other species of the genus Triti-cum, including spelt, as well as certain representatives of the family Poaceae [49]. Young spelt grass represents young green biomass formed during the early development of the plant. It is rich in minerals, vitamins, enzymes, phenolic compounds, and chlorophyll, which are responsible for its biological activity [50]. Due to its high germination capacity, even under unfavorable conditions, spelt can establish successfully during early stages of development and produce young grass rich in bioactive phyto-chemicals. This finding supports its use as functional foods, including spelt grass juice [51]. Evidence specifically addressing young spelt grass and spelt juice remains limited. Therefore, findings from common wheat and other cereals provide a useful comparative basis but cannot be directly extrapolated to spelt juice. Further studies are needed to establish optimal germination and pretreatment protocols for spelt, considering both the accumulation and potential loss of individual bioactive compounds, as well as their retention in the final juice.

### 4.2. Optimal Harvesting Stage of Young Spelt Grass

Given the limited number of studies on spelt grass and spelt juice, and considering the botanical relatedness of spelt and common wheat, the results of studies conducted on young cereals may serve as a basis for interpreting the physiological processes and phytochemical changes that occur during early plant development [38].

During germination and early development, cereals of the genus Triticum undergo a series of metabolic changes influencing synthesis, transformation, and accumulation of biloactive compounds [52]. Monitoring changes in the phytochemical composition enables the determination of the optimal harvesting period, i.e., the developmental stage at which the content of bioactive compounds is most favorable. Their composition depends on the growing conditions and developmental stage of the plant. Previous studies demonstrated that harvesting time significantly affects the nutritional and phytochemical profile of young biomass, as well as its antioxidant capacity [53,54].

Wheatgrass generally refers to the young shoots harvested during the early stages of development, commonly within approximately 6–10 days after germination, although the precise harvesting stage varies among studies and growing conditions [55,56]. The results of a recent study by Adhikari B. et al., examining the optimal harvesting period based on phytochemical content, implied the most favorable composition of young cereals during the ninth day of growth, particularly in phenolic compounds and their antioxidant potential [53]. It was shown that chlorophyll content, flavonoid content, and DPPH radical-scavenger activity were highest on day 9, whereas total phenolic content continued to increase until day 15. These findings indicate that the optimal harvesting time depends on the phytochemical parameter of interest. Although these results provide a useful reference for selecting the harvesting stage of young cereal grasses, further research is needed to establish the optimal harvesting time specifically for spelt grass intended for juice production [53,57].

### 4.3. Production of Spelt Juice

**Figure 1:**
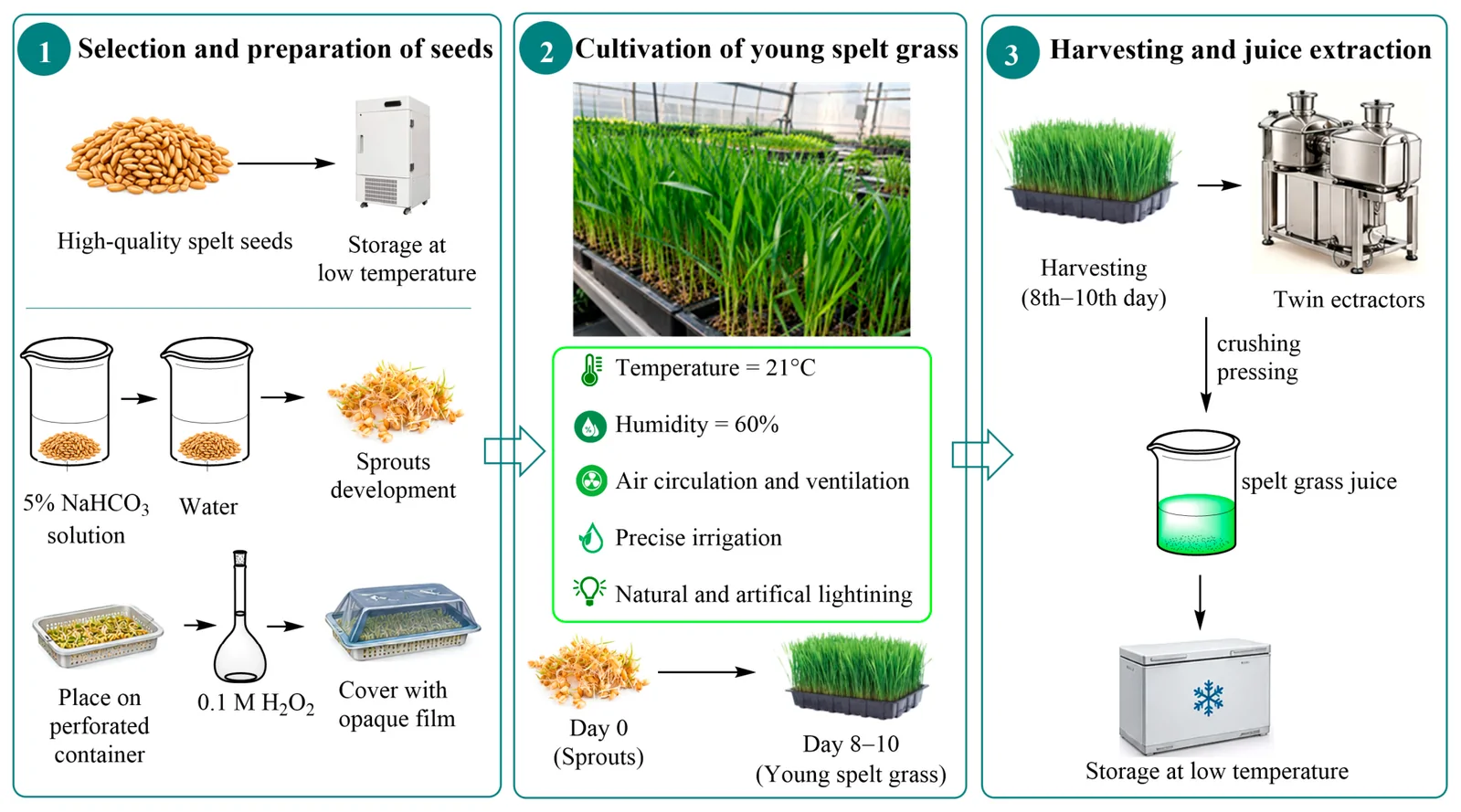
The production process of spelt juice.

Spelt juice is obtained from young shoots of spelt grass grown under specially controlled conditions, which influence the properties of the products obtained through their processing depend [12]. The production process of this beverage consists of three main stages: selection and preparation of seeds, cultivation of young spelt grass, and harvesting and juice extraction (Figure 1).

The first stage involves the adequate selection of high-quality seeds and their storage at low temperature to ensure efficient germination. The seeds are initially treated with a 5% NaHCO_3_ solution, followed by water in the first 36 h, during which they are periodically rinsed and drained, leading to the development of sprouts. The sprouts are then placed on a suitable perforated container, sprayed with a 0.1% H_2_O_2_ solution, and covered with an opaque film.

Young spelt grass is grown in a greenhouse under a controlled temperature of 21 °C and air humidity of 60%, with constant air circulation and ventilation, precise irrigation, and adequate lighting using a combination of natural and artificial light.

The final stage in the spelt juice production process involves harvesting the young grass between the 8th and 10th days after emergence, when the nutritional content of the plant reaches its peak. Immediately after harvesting, extraction is performed using twin extractors to preserve a greater number of enzymes and other bioactive compounds. Initially, special blades are used for crushing, followed by pressing, i.e., pressure extraction, during which spelt juice is obtained [58]. Due to its short shelf life, instability, and high perishability, the juice is stored at low temperatures, deeply frozen until further analysis or use. In order to achieve optimal product safety and stability, appropriate treatments such as freezing, high-pressure processing, ultraviolet irradiation, and ultrasonic treatment have been investigated to extend the shelf life of the juice and preserve its nutritional and therapeutic properties [59]. Freezing offers a relatively simple preservation option, while studies on wheatgrass juice have demonstrated the potential of alternative processing technologies to maintain product quality and extend shelf life [54,60]. Ultrasonication has shown potential to improve the microbiological quality of wheatgrass juice while preserving or increasing certain bioactive compounds. However, its effectiveness in microbial inactivation and its impact on heat-sensitive nutrients depend on the treatment conditions, warranting further investigation before its application to young spelt grass juice [54]. These findings suggest that suitable preservation methods could facilitate the wider availability and commercial use of young spelt grass juice. However, their economic feasibility and consumer acceptance require further evaluation.

## 5. Phytochemical Composition of Spelt Juice

**Figure 2:**
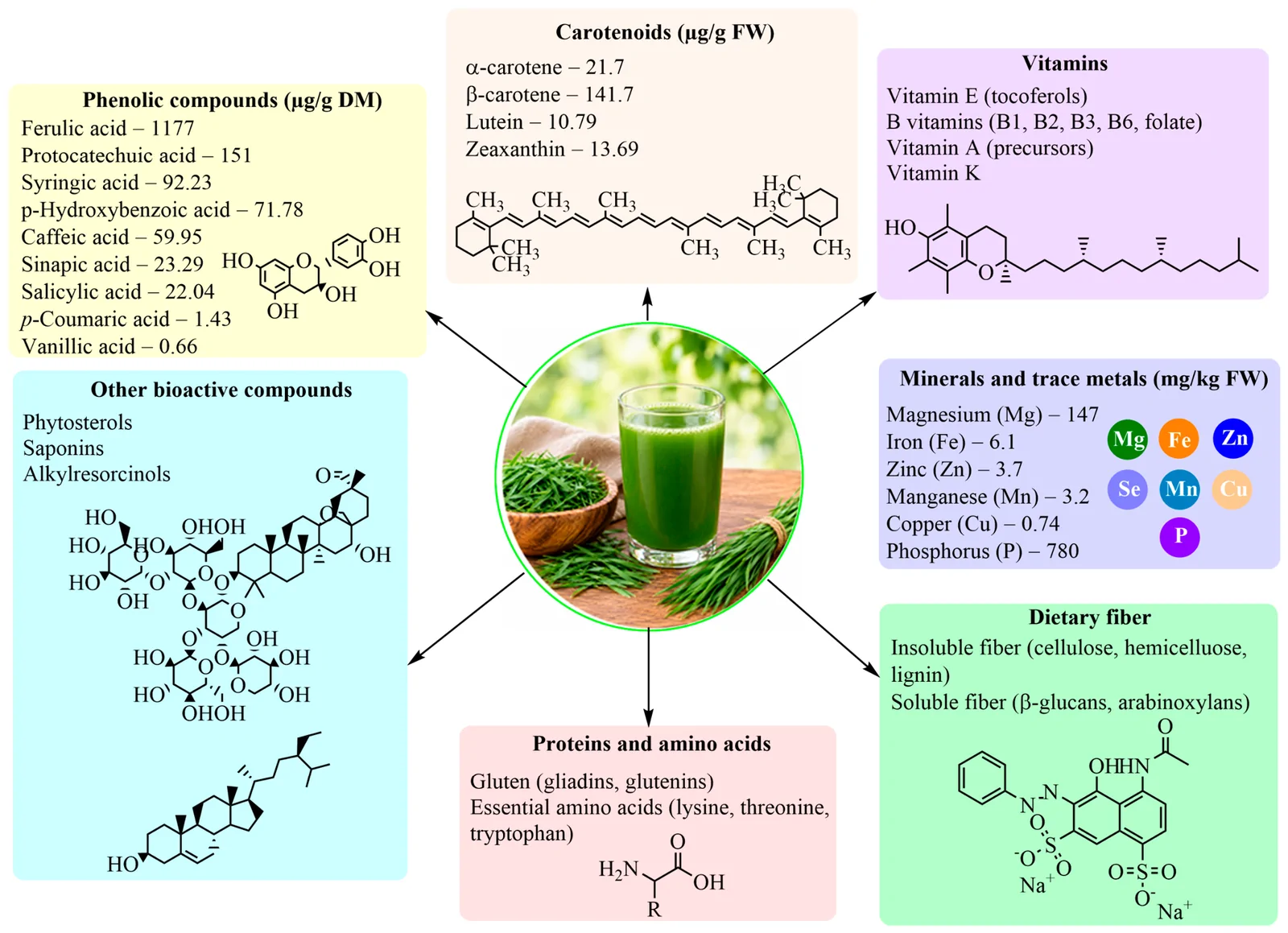
Phytochemical composition of young spelt grass extract and juice, including concentrations reported in the literature. DM—dry matter; FW—fresh weight.

Although data on spelt juice and its phytochemical composition are still limited, available studies indicate the presence of bioactive compounds exerting numerous therapeutic potentials. Young spelt grass extracts represent a significant source of nutrients and biologically active substances, including pigments (chlorophyll, xanthophylls, and carotenes), phenols, flavonoids, vitamins, and minerals [12]. The phytochemical composition of spelt juice is presented in Figure 2.

The growth, development, and phytochemical composition of young cereal grasses are also greatly influenced by growing conditions such as light, humidity, temperature, water availability, and soil quality [54,61]. Therefore, it is necessary to optimize the growth conditions of young spelt to obtain products with higher yield, improved phytochemical composition, and therapeutic potential.

### 5.1. Photosynthetic Pigments

Photosynthetic pigments are bioactive compounds that constitute an integral part of the photosynthetic apparatus. In addition to their primary role in the process of photosynthesis, they participate in photoprotective processes, thereby exerting antioxidant activity [62]. Chlorophylls, carotenoids, and phycobilins are the main photosynthetic pigments present in numerous plants, algae, and cyanobacteria [63].

Chlorophylls belong to the class of green pigments found in photosynthetic organisms and play a key role in the process of photosynthesis [64,65]. The most abundant photosynthetic pigments in plants are chlorophyll *a* and chlorophyll *b* [66]. It has been demonstrated that, in addition to their physiological function, they also exhibit antioxidant, anti-inflammatory, anticancer, antimutagenic, and anti-obesity properties [67].

Carotenoids are bioactive components responsible for the yellow and orange color of various fruits, flowers, and vegetables [68]. Carotenes and xanthophylls represent the two main groups of carotenoids, among which the most abundant are α-carotene, β-carotene, lutein, and zeaxanthin [69]. The primary role of carotenoids is the protection of photosynthetic apparatus, through dissipating excess light energy and neutralizing free radicals, thereby contributing to the protection of cells from oxidative stress. Accordingly, previous studies have confirmed their antioxidant, antitumor, anti-inflammatory, antidiabetic, and anti-aging properties [70].

Chlorophylls and carotenoids are present in young cereals as an integral part of their photosynthetic apparatus [71]. Previous studies of young spelt grass and products obtained through its processing have confirmed the presence of photosynthetic pigments. Additionally, chlorophyll *a*, chlorophyll *b*, carotenes, lutein, and zeaxanthin were identified in young spelt grass extracts and juice, and their content is presented in Table 2.

An earlier study determined the presence of chlorophyll *a*, chlorophyll *b*, α-carotene, β-carotene, xanthophylls, lutein and zeaxanthin in the young spelt grass extract. It was shown that the content of chlorophyll *b* was higher than that of chlorophyll *a*. However, it should be emphasized that part of chlorophyll *a* is converted into pheophytin *a* during the lyophilization process [12]. Moreover, Topalović et al. determined the presence of chlorophyll *a*, chlorophyll *b*, and total carotenoids in the extract of spelt juice and directly compared the obtained results with those found by Jančić et al. These groups of authors indicated that the contents of chlorophyll *a* and carotenoids were considerably higher, while the level of chlorophyll *b* was within a similar range. Despite differences in sample preparation, extraction methods, and analytical methods, both studies indicate the predominant presence of chlorophyll *a* and chlorophyll *b*, with a smaller amount of carotenoids [12,58].

### 5.2. Phenolic Compounds and Flavonoids

Phenolic compounds are secondary metabolites synthesized in plants under physiological conditions, but also in response to abiotic and biotic stress [72]. The main groups include phenolic acids, flavonoids, lignin, and stilbenes [73]. These compounds can neutralize free radicals, protect cells from oxidative damage, and exert antioxidant effects, potentially reducing the risk of cardiovascular, malignant, metabolic, and neurodegenerative diseases [74,75].

Cereals, including spelt, are a significant source of phenolic compounds, particularly phenolic acids and flavonoids, whose content and composition depend on genetic factors, environmental conditions, and processing methods [76,77]. However, their composition in young spelt grass and spelt juice has been investigated in a limited number of studies, summarized in Table 3 [12,58].

Jančić et al. reported the highest total phenolic and flavonoid contents in young spelt grass harvested on the ninth day, with aqueous extracts showing higher values than ethanolic extracts [12]. Regarding the individual phenolic compounds in spelt juice, chromatographic analysis by Topalović et al. identified phenolic acids as the predominant compounds, with ferulic acid being particularly prominent. Its concentration exceeded 1100 µg/g of dry matter, accounting for approximately one-third of the total analyzed phenolics. Protocatechuic acid was another significant compound, reaching 150 µg/g of dry matter, followed by syringic, salicylic, and sinapic acids and their derivatives [58].

In addition to photosynthetic pigments and phenolic compounds, young spelt grass extracts have a rich nutritional composition, reflected, among other things, in the presence of various minerals. In their results, Jančić et al. indicated a significant content of potassium, phosphorus, manganese, and chromium, whereby 100 g of fresh extract provides 7.5–16% of the reference daily intake of these elements [12].

### 5.3. Comparison of the Phytochemical Composition of Spelt and Wheat Juice

Juices obtained by processing cereals have gained importance in recent years as beverages with therapeutic and preventive potential, although their molecular and cellular mechanisms and phytochemical composition have not yet been fully elucidated [78]. Wheatgrass juice possesses numerous bioactive components that contribute to its therapeutic properties [79]. Due to limited research on the phytochemical composition of spelt juice, available findings can be compared with those on wheatgrass juice, an agronomically and morphologically related species.

Previous studies have identified similarities between young spelt grass and wheatgrass, as well as differences related to genotype and growing conditions. Popović et al. compared three spelt genotypes and one wheat genotype and found that organically grown spelt Ebners–Rotkorn had the highest antioxidant capacity, reflected in total phenolic content, superoxide dismutase activity, DPPH antiradical power, and FRAP, alongside lower lipid peroxidation [80]. These findings indicate the antioxidant potential of spelt grass and support further research into its phytochemical composition and processed products.

Table 4 summarizes available data for comparing selected phytochemical components of spelt and wheat juice. Their composition in terms of photosynthetic pigments and phenolic compounds appears similar, although the reported contents differ between studies. Spelt juice analyzed by Topalović et al. showed high total chlorophyll and carotenoid contents. However, phenolic compounds were measured in spelt juice extracts, preventing direct quantitative comparison with wheat juice. Nevertheless, phenolic compounds are important constituents of both beverages, with ferulic acid predominating in spelt juice and catechin reported as the predominant compound in wheat juice by Skoczylas et al. [58,61,81].

## 6. Therapeutic Potential of Spelt Juice

**Figure 3:**
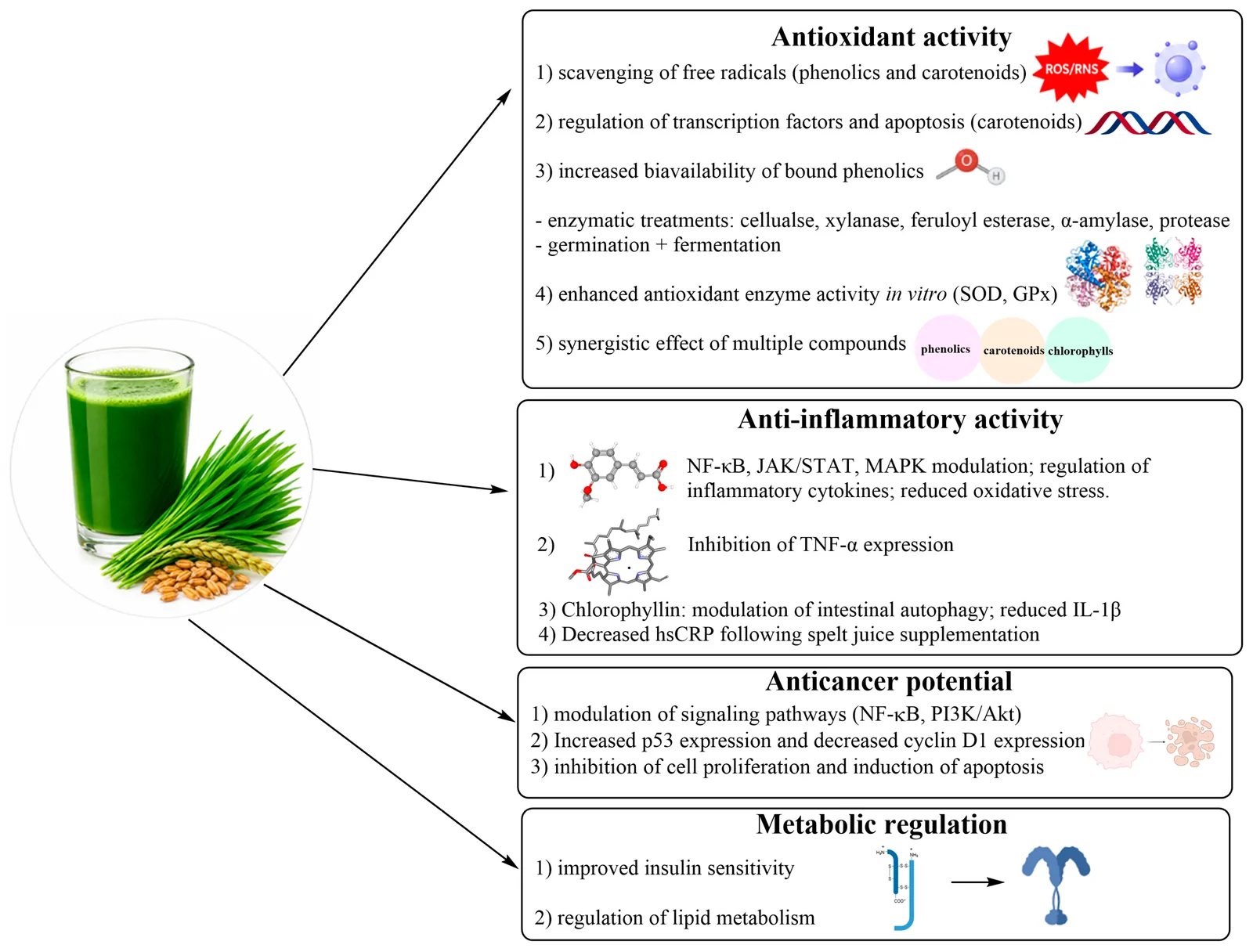
Potential mechanisms underlying the biological activities of spelt grass juice.

Considering the biological effects of all phytochemical components, young spelt grass and spelt juice represent functional foods with significant potential for application in the prevention of various disorders and the maintenance of overall health, exerting antioxidant, anti-inflammatory, antiproliferative, and metabolic effects [12,58]. Potential therapeutic effects of young spelt juice are presented in Figure 3.

### 6.1. Antioxidant Activity

Oxidative stress is defined as an imbalance between prooxidants and antioxidants in favor of prooxidants, whereby increased production of reactive oxygen and nitrogen species (ROS and RNS) may lead to molecular damage and disruption of redox signaling [82]. The antioxidant defense system plays a crucial role in maintaining cellular homeostasis. It is activated in response to excessive free radical production and helps prevent oxidative damage to cellular components [83]. Natural antioxidants found in fruits, vegetables, and cereals, including vitamins, phenolic compounds, anthocyanins, carotenoids, and others, play an important role in disease prevention [84]. Phenolic compounds and photosynthetic pigments, which act as antioxidants in spelt juice, contribute substantially to its overall antioxidant potential. Phenolic compounds serve as electron or hydrogen atom donors, thus neutralizing free radicals [85]. In addition to their ability to neutralize free radicals, carotenoids regulate the activity of transcription factors and influence the process of cell apoptosis, which together enhances the protection of cells against oxidative damage [86].

A previously conducted study examined six spelt varieties to determine the antioxidant potential of this beverage, using eight methods based on different mechanisms. The results showed that phenolic acids were the most abundant class of phenolic compounds, with their composition and concentrations varying among the varieties examined. It was suggested that bound forms of phenolic acids possess greater antioxidant activity. However, by analyzing the antioxidant potential, this study concluded that free phenolic compounds were more effective to prevent disturbance of redox state [87]. The same finding regarding the availability of phenolic compounds was also demonstrated by Mencin et al. They highlighted the importance of enzymatic treatments as a method for releasing bound phenolic compounds from spelt seeds. The combination of different enzymes, such as cellulase, xylanase, feruloyl esterase, α-amylase, and protease, leads to increased availability and antioxidant activity of free *p*-coumaric, ferulic, and *p*-hydroxybenzoic acids, with particularly pronounced activity in the 2,2′-azinobis(3-ethylbenzothiazoline-6-sulfonic acid) (ABTS•+) assay [88].

Moreover, germination represents an important process in increasing the antioxidant potential of spelt. During the germination process, changes occur in the composition of secondary metabolites in spelt grain. These changes are reflected in an increase in content of phenolic compounds and antioxidant activity [40]. Emphasis is placed on the combination of enzymatic treatments with simultaneous germination and fermentation. This combined method results in an increase in the content of trans-ferulic acid, which is identified as a phenolic compound that may protect against pathological conditions caused by oxidative stress, including cancer, cardiovascular, and neurodegenerative diseases [88].

It was earlier mentioned that antioxidant activity directly depends on the spelt genotype. Additionally, Jančić et al. showed that the antioxidant capacity of young spelt grass extract also depends on growing conditions. It was confirmed that extracts obtained by young spelt grass harvested on the 9th day after germination, under adequate lighting and irrigation, showed the highest antioxidant activity [12,80]. The antioxidant potential determined by Jančić and colleagues was positively associated with the content of polyphenols and flavonoids, as well as photosynthetic pigments such as chlorophyll *a*, chlorophyll *b*, pheophytin, and carotenoids, which additionally enhance the antioxidant activity of these extracts [12]. As we previously mentioned, there is little evidence examining antioxidant potential of young spelt grass extract on animal or human models. Both available studies determining the antioxidant potential of young spelt grass used an in vitro model to determine the antioxidant enzymes including superoxide dismutase and guaiacol peroxidase, as well as ABTS, DPPH, and FRAP assays [12,80].

A recent study on spelt juice confirmed that young spelt grass is a significant source of phenolic compounds, among which ferulic acid stands out as the dominant component, as well as photosynthetic pigments that exert a synergistic antioxidant effect and contribute to the biological activity of spelt juice [58].

Although the antioxidant capacity of spelt grass and spelt juice has been investigated using chemical assays, evidence from cell-based and animal studies remains limited. Nevertheless, some evidence of potential antioxidant effects is available from human studies. Antonić et al. monitored oxidative stress parameters in twenty participants following two months of daily consumption of spelt juice. The results of the conducted study showed significantly decreased PAB (prooxidant–antioxidant balance) and AOPP (advanced oxidation protein products) values, parameters indicative of reduced oxidative stress, while TAS (total antioxidant status) and SH group concentrations increased, indicating an improvement in overall antioxidant capacity. Based on the analysis of these results, it can be concluded that spelt juice may have beneficial effects on oxidative stress parameters. However, caution should be exercised when interpreting the results because this clinical study had limitations in terms of the small number of participants and study duration, and therefore well-controlled clinical trials are necessary [89].

### 6.2. Anti-Inflammatory Activity

Inflammation represents a physiological defense mechanism triggered by chemical, mechanical, and microbial tissue damage and aims to prevent further damage and promote the regeneration of damaged tissue [90]. In recent years, oxidative stress has often been associated with inflammatory processes, as free radicals may participate in the activation of inflammatory signaling pathways, thereby triggering a cellular adaptive response [91]. Since inflammation plays an important role in the development of many diseases, biologically active compounds naturally present in foods may contribute to the reduction in inflammatory processes [92].

The bioactive components of spelt juice, primarily phenolic compounds and chlorophyll, may represent potential modulators of the inflammatory response. As previously mentioned, the most abundant phenolic compound in spelt juice is ferulic acid [58]. Previous studies have shown that ferulic acid may exert anti-inflammatory effects through various mechanisms, including modulation of signaling pathways such as nuclear factor kappa B (NF-κB), Janus kinase/signal transducer and activator of transcription (JAK/STAT), and mitogen-activated protein kinase (MAPK), regulation of inflammatory cytokine levels, regulation of immune cell homeostasis, and inhibition of oxidative stress, thereby also contributing to improved antioxidant protection [93]. Chlorophyll, the main photosynthetic pigment in spelt juice, has also exhibited potential anti-inflammatory properties in experimental models. On the one hand, chlorophyll *a* directly inhibits the expression of the gene encoding the pro-inflammatory cytokine tumor necrosis factor-alpha (TNF-α). On the other hand, the results of a study conducted using an experimental model of inflammatory bowel disease showed that chlorophyllin, a derivative of chlorophyll, may affect the process of autophagy in the intestine and leads to reduced levels of interleukin-1 beta (IL-1β) and TNF-α, thereby reducing intestinal inflammation [94,95].

Direct evidence of the anti-inflammatory effects of spelt grass juice remains limited. However, Antonić et al. (2021) reported a statistically significant reduction in high-sensitivity C-reactive protein (hsCRP) concentrations following two months of daily spelt juice consumption by 20 volunteers (*p* < 0.001). Before supplementation, 25.0% of participants had hsCRP levels associated with high cardiovascular risk, whereas after supplementation, 47.7% had levels associated with low risk and only 10.5% remained in the high-risk category. These findings suggest a potential anti-inflammatory effect of spelt grass juice, possibly related to its bioactive constituents, including chlorophyll, although this mechanism remains to be confirmed. The observed changes in inflammatory status were accompanied by improvements in oxidative stress and antioxidant status markers, as described above [89].

### 6.3. Antiproliferative Activity

Plant medicines, as well as products obtained by processing plants, act as promising agents in cancer therapy due to their diverse chemical structures, phytochemical composition, and biological activity. They possess a therapeutic advantage related to selective toxicity toward cancer cells with reduced adverse effects associated with conventional chemotherapy [96]. Plant secondary metabolites, particularly phenolic compounds and flavonoids, have attracted increasing interest due to their potential to regulate numerous signaling pathways in cancer. As phytochemicals, these compounds may reduce cell proliferation by inhibiting NF-κB, cyerclin *D*, cyclin-dependent kinases (CDK), extracellular signal-regulated kinase (ERK1/2), oncogenic signaling cascades (*PI3K* and *Akt*), angiogenic factors (VEGF and MIC-1), apoptosis, and inhibition of cell migration and metastasis [97]. Taking all of these findings, plant-derived phenolic compounds need to be further studied due to their potential in both cancer prevention and treatment [98].

Considering that spelt extracts contain high levels of phytochemicals, their antiproliferative potential has been investigated in various in vitro studies on human tumor cell lines. Ahmed et al. investigated aqueous, 50% ethanolic, and 100% ethanolic extracts of spelt on HepG2 liver cancer cells and Caco-2 colorectal cancer cells. The highest activity was observed for the aqueous extract, with IC_50_ values of 226.4 μg/mL for HepG2 and 247.6 μg/mL for Caco-2 cells. Apoptotic response and the expression of genes involved in proliferation, invasion, and angiogenesis were also investigated, resulting in increased expression of p53, along with reduced expression of Cyclin D1. This may contribute to the inhibition of tumor cell proliferation [99]. Antiproliferative and apoptotic effects of spelt extract were further investigated by the same group of authors. In this research they used human tumor cells of the lung (A549), breast (MDA-MB 231), and liver (Huh7) and obtained interesting results which confirmed a pronounced antiproliferative effect accompanied by an increase in the apoptotic response of examined extracts. These effects may be associated with the phenolic compounds present in extract, including vanillic, gallic, and protocatechuic acids and pyrocatechol. Moreover, it was assumed that these compounds could modulate NF-κB and nuclear factor erythroid 2-related factor 2 (Nrf2) signaling pathways [100].

The most significant results related to the antiproliferative activity of extracts obtained directly from young spelt grass juice were reported in the study by Topalović et al. It was shown that young spelt extracts showed a moderate antiproliferative effect on human A549 lung cancer cells and HeLa cervical cancer cells, while simultaneous administration of the extract with doxorubicin further increased the cytotoxic effect. These effects largely depend on the choice of solvent. In addition, acetone–water extract, in comparison with the aqueous and methanol–water extracts, showed the highest selectivity toward HeLa cells. This finding may be explained by the ability of these solvents to extract phenolic compounds and photosynthetic pigments, as key phytochemical components that may modulate oncogenic pathways [58]. Previous studies have shown that ferulic acid exerts anticancer potential by inhibiting proliferation, invasion, metastasis, and angiogenesis of tumor cells, as well as by modulating enzyme activity, gene expression, and signaling pathways [101]. In addition to ferulic acid, chlorophyll and its derivatives also play an important role, as they may inhibit tumor cell proliferation, which is associated with changes in the redox status of cells and the induction of apoptosis [102]. Although these results were not obtained by investigating spelt juice, they indicate a possible contribution of ferulic acid and chlorophyll to its antiproliferative potential, which requires further investigation.

### 6.4. Metabolic Activity

In addition to the previously described biological activities of spelt, the products obtained by this plant may expose potential effects on metabolism, particularly on glucose and lipids, due to its phytochemical composition. The high amount of dietary fiber, as well as the presence of phytic acid and alkylresorcinols in spelt grain, may contribute to better blood glucose control and insulin sensitivity, while antioxidant compounds in spelt grain exert a protective effect in cases of chronic hyperglycemia [103].

In an in vivo study conducted on a model of rats with induced type 2 diabetes mellitus, a spelt-based diet led to a reduction in regulatory genes involved in lipid and glucose metabolism, as well as a low acute glycemic response compared with wheat [104]. Evidence of the hypoglycemic effects of young spelt grass and spelt juice is still limited. However, the results of studies examining the biological activity of young wheatgrass indicate the potential of young cereal grass, including young spelt grass, on glucose metabolism. In an experimental model of diabetes, administration of wheatgrass positively affected changes in insulin and glycogen levels, as well as changes in the activity of enzymes involved in glucose metabolism, indicating a significant hypoglycemic role of wheatgrass [105].

The results of the study by Antonić et al., investigating the effect of spelt juice on lipid metabolism, did not show significant changes in the main lipid profile parameters (LDL-C, HDL-C, total cholesterol, triglycerides). On the other hand, it was observed that administration of spelt juice reduced coronary risk and the atherogenic index, and increased campesterol level, a lipid homeostasis marker. These results indicate that supplementation with spelt juice has a positive effect on the lipid profile [89]. Given the limited amount of data, further studies are needed to additionally investigate and confirm the metabolic effects of spelt juice.

## 7. Limitations and Future Research Directions

Despite the therapeutic potential, research on spelt juice remains limited. The limitations of these studies include the lack of studies conducted in humans, while the available data have been obtained mostly from in vitro studies. Furthermore, the results of the studies should be interpreted with caution, since some of them concern whole seeds, extracts, or young spelt grass and therefore cannot be directly compared or equated with the biological properties of spelt juice. An additional challenge is the standardization of the composition and production methods of spelt juice, which may complicate the comparison of results from different studies. Therefore, further research directly focused on spelt juice is necessary, particularly studies involving a larger number of participants, to investigate in detail its phytochemical composition, exact mechanisms of action, as well as its potential application in the prevention and treatment of various diseases.

## 8. Conclusions

Based on the available literature, it can be concluded that spelt juice stands out as a nutritionally valuable functional food rich in biologically active compounds, with pronounced therapeutic potential. Phenolic compounds and photosynthetic pigments, as the main phytochemical components, contribute to the antioxidant, anti-inflammatory, antiproliferative, and metabolic effects of spelt juice. Although studies linking the consumption of spelt juice to its therapeutic potential remain limited, they indicate promising pathways for its potential application in disease prevention and health promotion. Despite gaps in the literature, the diverse spectrum of reported positive effects of spelt juice supplementation provides insight into its possible contribution to various physiological processes and encourages further research into its biological effects. Therefore, these results may contribute to expanding knowledge about spelt juice as a functional food, increase interest, and serve as a basis for future research into its mechanisms of action and potential application within a healthy diet and the prevention and treatment of various diseases.

## Figures and Tables

**Table 1:** Comparison of the nutritional composition of spelt and common wheat.

Cereal	Protein (%)	Lipids (%)	Total Dietary Fiber (%)	Insoluble Fiber (%)	Iron (mg/kg)	Zinc (mg/kg)
Spelt	15.6 [29], 16.0 [33]	2.5 [29]	10.9 [29]	9.3 [29]	45.63 [34]	42.31 [34]
Common wheat	14.9 [29], 12.2 [33]	2.1 [29]	14.9 [29]	11.2 [29]	32.63 [34]	32.79 [34]

**Table 2:** Photosynthetic pigment content in young spelt grass and spelt juice.

Sample/Reference	Chlorophyll *a*	Chlorophyll *b*	Total Carotenoids *
Young spelt grass extract [12]	2.402–7.077 mg/100 g FW	8.550–28.096 mg/100 g FW	2.45–3.08 mg/100 g FW
Spelt juice [58]	≈40 mg/100 g FW	≈15 mg/100 g FW	≈10 mg/100 g FW
Extract of lyophilized spelt juice [58]	6.39 ^1^–6.91 ^2^ mg/g DW	2.47 ^1^–2.69 ^2^ mg/g DW	1.81 ^1^–1.95 ^2^ mg/g DW

FW—fresh weight, DW—dry weight, *—sum of the mean values for the individual identified carotenoids, ^1^—lyophilized samples of spelt grass juice without foam, ^2^—lyophilized samples of spelt grass juice with foam.

**Table 3:** Content of phenolic compounds and flavonoids in young spelt grass and spelt juice.

Sample/Reference	Parameter	Content
Young spelt grass extract [12]	Total phenolic content (TPC)	39.7–118.7 mg GAE/g FW
Young spelt grass extract [12]	Total flavonoid content (TFC)	8.5–129.0 mg CE/g FW
Spelt juice [58]	Total phenolics *	2685–3601 µg/g DW

FW—fresh weight, DW—dry weight, GAE—gallic acid equivalents, CE—catechin equivalents, *—sum of 22 compounds.

**Table 4:** Phytochemical Profile of Spelt and Wheat Juice.

Sample/Reference	Total Chlorophylls	Total Carotenoids	Total Phenolics
Spelt juice [58]	≈550 mg/L	≈110 mg/L	2685–3601 µg/g DW *
Wheat juice [81]	391.4 mg/L	79.1 mg/L	920.9 mg CE/L
Wheat juice [61]	254–279 mg/L	23–29 mg/L	289–342 mg GAE/L

DW—dry weight, GAE—gallic acid equivalents, CE—catechin equivalents, *—total phenolics determined in spelt juice extracts.

## Data Availability

No new data were created or analyzed in this study. Data sharing is not applicable to this article.
